# Community Perspectives of Complex Trauma Assessment for Aboriginal Parents: ‘Its Important, but *How* These Discussions Are Held Is Critical’

**DOI:** 10.3389/fpsyg.2020.02014

**Published:** 2020-09-15

**Authors:** Catherine Chamberlain, Graham Gee, Deirdre Gartland, Fiona K. Mensah, Sarah Mares, Yvonne Clark, Naomi Ralph, Caroline Atkinson, Tanja Hirvonen, Helen McLachlan, Tahnia Edwards, Helen Herrman, Stephanie J. Brown, and Jan M. Nicholson

**Affiliations:** ^1^Judith Lumley Centre, La Trobe University, Melbourne, VIC, Australia; ^2^NGANGK YIRA: Murdoch University Research Centre for Aboriginal Health and Social Equity, Perth, WA, Australia; ^3^Intergenerational Health Group, Murdoch Children’s Research Institute, Melbourne, VIC, Australia; ^4^School of Psychology, The University of Melbourne, Melbourne, VIC, Australia; ^5^Department of Pediatrics, The University of Melbourne, Melbourne, VIC, Australia; ^6^Clinical Epidemiology and Biostatistics Unit, Murdoch Children’s Research Institute, Melbourne, VIC, Australia; ^7^Royal Children’s Hospital, Melbourne, VIC, Australia; ^8^School of Psychiatry, University of New South Wales, Sydney, NSW, Australia; ^9^College of Medicine and Public Health, Flinders University, Darwin, NT, Australia; ^10^SAHMRI Women and Kids Theme, South Australian Health and Medical Research Institute, Adelaide, SA, Australia; ^11^School of Psychology, University of Adelaide, Adelaide, SA, Australia; ^12^We Al-li Pty Ltd, Goolmangar, NSW, Australia; ^13^Central Australian Aboriginal Congress, Alice Springs, NT, Australia; ^14^Orygen, National Centre of Excellence in Youth Mental Health, University of Melbourne, Melbourne, VIC, Australia; ^15^Centre for Youth Mental Health, The University of Melbourne, Melbourne, VIC, Australia; ^16^Population Health, Murdoch Children’s Research Institute, Melbourne, VIC, Australia

**Keywords:** Aboriginal, indigenous, culture, complex trauma, parent, assessment

## Abstract

**Background and Purpose:**

Becoming a parent can be an exciting and also challenging transition, particularly for parents who have experienced significant hurt in their own childhoods, and may be experiencing ‘complex trauma.’ Aboriginal and Torres Strait Islander (Aboriginal) people also experience historical trauma. While the parenting transition is an important time to offer support for parents, it is essential to ensure that the benefits of identifying parents experiencing complex trauma outweigh any risks (e.g., stigmatization). This paper describes views of predominantly Aboriginal stakeholders regarding (1) the relative importance of domains proposed for complex trauma assessment, and (2) how to conduct these sensitive discussions with Aboriginal parents.

**Setting and Methods:**

A co-design workshop was held in Alice Springs (Central Australia) as part of an Aboriginal-led community-based participatory action research project. Workshop participants were 57 predominantly Aboriginal stakeholders with expertise in community, clinical, policy and academic settings. Twelve domains of complex trauma-related distress had been identified in existing assessment tools and through community consultation. Using story-telling and strategies to create safety for discussing complex and sensitive issues, and delphi-style methods, stakeholders rated the level of importance of the 12 domains; and discussed why, by whom, where and how experiences of complex trauma should be explored.

**Main Findings:**

The majority of stakeholders supported the importance of assessing each of the proposed complex trauma domains with Aboriginal parents. However, strong concerns were expressed regarding where, by whom and how this should occur. There was greater emphasis and consistency regarding ‘qualities’ (e.g., caring), rather than specific ‘attributes’ (e.g., clinician). Six critical overarching themes emerged: ensuring emotional and cultural safety; establishing relationships and trust; having capacity to respond appropriately and access support; incorporating less direct cultural communication methods (e.g., yarning, dadirri); using strengths-based approaches and offering choices to empower parents; and showing respect, caring and compassion.

**Conclusion:**

Assessments to identify Aboriginal parents experiencing complex trauma should only be considered when the prerequisites of safety, trusting relationships, respect, compassion, adequate care, and capacity to respond are assured. Offering choices and cultural and strengths-based approaches are also critical. Without this assurance, there are serious concerns that harms may outweigh any benefits for Aboriginal parents.

## Introduction

Becoming a parent is an exciting time, but it can also be a challenging transition, particularly for parents who have experienced maltreatment in childhood and continue to experience complex trauma. There are compelling arguments to recognize and offer support to parents who have experienced childhood maltreatment and/or complex trauma (Flanagan et al., 2018). However, essential criteria specify that any screening or assessment process must be acceptable to the population and that the benefits outweigh the harms (Wilson and Jungner, 1968; Department of Health, 2018). This is particularly important for Aboriginal parents following a legacy of harmful policies which have led to family disruption and increased rates of intergenerational or complex trauma; and fostered an environment which is highly sensitive about assessment of complex trauma, particularly within perinatal care services. As far as we are aware there are no studies reporting perspectives of Aboriginal communities regarding perinatal assessment of childhood maltreatment or complex trauma (Chamberlain et al., 2019b). In this paper, we report on an innovative combination of Aboriginal leadership, participatory design and methods to elicit the perspectives of key stakeholders working with Aboriginal parents regarding complex trauma assessment in the perinatal period.

Up to 50% of all children worldwide experience maltreatment (World Health Organisation [WHO], 2016) with potentially profound and enduring effects on physical, social and emotional wellbeing (McCrory et al., 2010; Norman et al., 2012; De Bellis and Zisk, 2014; Alexander, 2015; Li et al., 2016; Teicher and Samson, 2016; Sara and Lappin, 2017; Olsen, 2018). Critically, the long-term relational impacts can impede the capacity of parents to nurture and care for their own children (Alexander, 2015). Increasing evidence regarding these ongoing psychological effects has led to evolving international consensus to formalize recognition of a cluster of symptoms as ‘complex post-traumatic stress disorder (complex trauma)’ (Maercker et al., 2013; Bremness and Polzin, 2014; Cloitre et al., 2014). This symptom cluster includes *affect/emotional dysregulation*, *negative self-concept* and *relational disturbances*, in addition to the previously recognized post-traumatic stress disorder (PTSD) symptoms of *re-experiencing the events (triggers)*, *avoidance*, and a *sense of threat* (Maercker et al., 2013; Cloitre et al., 2014).

The perinatal period (pregnancy to 2 years after birth) is a critical life-course transition (Marmot et al., 2012) for parents who experience complex trauma (Grote et al., 2012). Trauma responses may be ‘triggered’ during pregnancy, birth and breastfeeding (Dierkhising et al., 2013) and while caring for a baby (Amos et al., 2011). Trauma responses may cause confusion and compound parental distress, as the original trauma may have occurred much earlier in life and not be consciously linked to their current experience. The parenting transition offers a unique opportunity for emotional development and recovery (Violence Prevention Alliance, 2016; Chamberlain et al., 2019a). For many who have experienced childhood maltreatment, becoming a parent is an opportunity for a ‘fresh start’ (Chamberlain et al., 2019c). A ‘hope-inspiring’ focus can transform the ‘vicious cycle’ of intergenerational trauma into a ‘virtuous cycle,’ positively reinforcing nurturing, competence and love (Atkinson et al., 2010). Frequent scheduled contacts with healthcare providers before and after childbirth offer an opportunity for recognition of complex trauma and provision of support, often being the first time since childhood this predominantly young childbearing population have had regular contact with healthcare services. This period is also a critical time to enhance early parenting relationships and prevent transmission of trauma (Atkinson et al., 2010; Scott, 2013; O’Donnell et al., 2019). Thus there are calls for health and mental health screening, including identifying parents who have experienced childhood maltreatment, to be included as a routine part of perinatal care in Australia (Austin et al., 2013; Rahman et al., 2013).

There is currently limited evidence regarding the impacts of complex trauma within Aboriginal and Torres Strait Islander (Aboriginal) communities. However, Aboriginal communities have identified historical and intergenerational trauma associated with colonization as a critical factor impacting on health (Atkinson, 2008; Gee, 2016). Increasing international evidence suggests that complex trauma could be potentially responsible for a significant proportion of the stark inequities in health experienced by Aboriginal people (Australian Institute of Health, and Welfare, 2014). Effective strategies to address these inequities are urgently needed (Australian Government, 2019), and support during the perinatal period is likely to have a higher impact than any other period in the life-course (Marmot et al., 2012). In discussion with key stakeholders working with Aboriginal parents during the perinatal period we aimed to examine their perspectives regarding recognition and assessment of complex trauma, specifically to answer the following questions:

1. What is the relative importance of proposed *key domains* that could be included in *assessment* of Aboriginal parents experiencing complex trauma?
2. What are the *key prerequisites* for ensuring *safe recognition* of Aboriginal parents experiencing complex trauma in the perinatal period? i.e., why, by whom, where and how should issues related to complex trauma be discussed?

## Materials and Methods

### Project Context and Methodology

The Healing the Past by Nurturing the Future (HPNF) project is an Aboriginal-led community-based participatory action research (action research) project and is described in detail elsewhere (Chamberlain et al., 2019a). Indigenous research methodologies underpin the conceptual framework for the overall HPNF project and the specific study outlined in this manuscript. Indigenist research methodologies recognize ongoing oppression, and transgenerational trauma, grief and loss for Aboriginal communities and aim to assist decolonization through a process of empowering and privileging Aboriginal worldviews and self-determination (Rigney, 2006).

**FIGURE 1:**
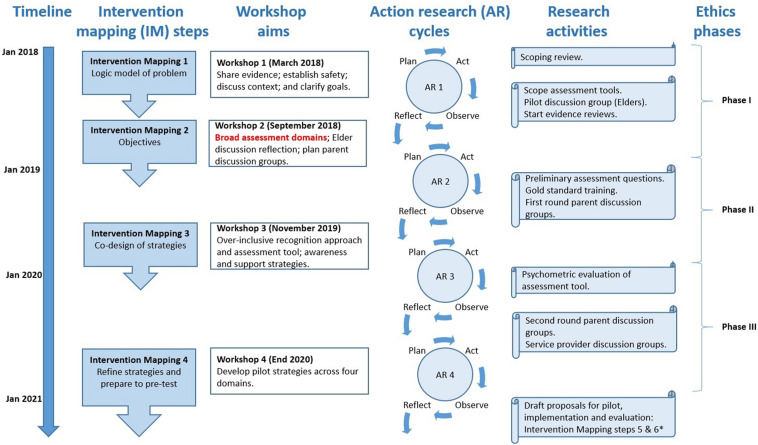
Context of study within Healing the Past by Nurturing the Future project.

The HPNF project aims to co-design perinatal awareness, recognition, assessment and support strategies for Aboriginal parents experiencing complex trauma using an Intervention Mapping framework (Bartholomew et al., 2016). The HPNF project is being conducted in three Australian jurisdictions (Northern Territory, South Australia, and Victoria) with predominantly Aboriginal key stakeholders from all state and territory jurisdictions participating in co-design workshops with the Aboriginal-led research team. Four action research cycles incorporate mixed methods research activities, which include meaningful participative processes with Aboriginal parents, and reflection and planning stages for research activities conducted in four key stakeholder co-design workshops. Workshops are aligned with the first four (of six) Intervention Mapping steps which are being conducted in the currently funded HPNF project. Future grant proposals will be submitted for Intervention Mapping steps 5 and 6 (implementation and evaluation) following completion of these first four co-design steps. Thus, each co-design workshop has specific goals, working progressively in a step-wise approach toward development of context-relevant strategies. The *research study* described in this manuscript was conducted within the second *HPNF project co-design workshop*, and illustrated in Figure 1.

### Information From the Previous HPNF Project Action Research Cycle That Has Informed This Current Research Study

Participants attending the first HPNF co-design workshop expressed concern about potential risks of recognition of parents experiencing complex trauma. In particular, they identified the potential for inappropriate referral to child protection services (Ralph et al., 2018). Aboriginal people have demonstrated a continued resilience to harmful legacies of colonization, including violence and historical trauma, oppression and state-sanctioned systematic removal of Aboriginal children from their families (‘stolen generation’) (Atkinson et al., 2010), which has impacted on social, emotional and physical wellbeing (Evans-Campbell and Walters, 2006; Sotero, 2006). While there are many encouraging stories; there are currently also ongoing efforts to re-unify children from the ‘stolen generation’ with families, and disturbing reports of high rates of child maltreatment and removal of children from families in some communities (O’Donnell et al., 2019). These cumulative issues have fostered a highly sensitive environment for identifying and addressing complex trauma within Aboriginal communities. This is particularly so for perinatal care services that are strongly implicated in historical and ongoing removal of Aboriginal children from families (Social Justice Commissioner, 2007). Current discourse around ‘intergenerational cycles of maltreatment’ compounds concerns about the potential for service providers to assume all parents with a history of child maltreatment are likely to maltreat their own children and involve child protection services based on erroneous and ill-informed assumptions.

Concerns were also expressed regarding ‘labeling’ parents as ‘having problems’ and undermining resilience and coping skills. Previous workshop participants discussed the importance of strengths-based approaches to avoid reinforcing the dominant negative discourse regarding Aboriginal people in Australia (Fogarty et al., 2018), much of which has evolved from research from which Aboriginal people have been largely excluded. The lives of Aboriginal families have often been viewed through the lens of non-Aboriginal researchers as the ‘problematic other’ (Laycock et al., 2011).

Another challenge discussed was the relevance of international complex trauma constructs for Aboriginal people. While there is growing consensus on the symptom clusters associated with ‘complex trauma’ (Cloitre et al., 2014; World Health Organisation [WHO], 2018), there are still no specified diagnostic criteria (Jowett et al., 2019), and there is no ‘gold standard’ measure, including in relation to culturally and linguistically diverse communities. In addition, the 11th edition of the International Statistical Classification of Diseases and Related Health Problems (World Health Organisation [WHO], 2018) (ICD11) symptom clusters do not include domains or ‘areas of distress’ that are necessarily aligned with Aboriginal understandings of social and emotional wellbeing (Social Policy Research Centre, and Nulungu Research Centre, 2013; Commonwealth of Australia, 2017) or previous research that has identified other potentially important cultural idioms of distress (Atkinson, 2008). Family, kinship and connectedness are a central tenet of Aboriginal understandings of social and emotional wellbeing (Gee et al., 2014) and community cohesion, access to services and cultural continuity have demonstrated a protective effect for some trauma-related outcomes among Aboriginal peoples (Chandler and Lalonde, 2008).

### Identifying and Synthesizing Existing Complex Trauma Assessment Tools

Prior to this second co-design workshop, the project Assessment Working Group reviewed over 25 assessment tools. These were identified from a scoping review of assessment tools currently used in the perinatal period (Chamberlain et al., 2019b), and from consultation with leaders in the field. Information about the assessment tools were collated, with items mapped onto existing trauma symptom clusters (American Psychiatric Association [APA], 2013; Cloitre et al., 2014; Böttche et al., 2018; World Health Organisation [WHO], 2018), and additional Aboriginal cultural idioms of distress (Atkinson, 2008). The working group members determined that a domain regarding ‘recognition’ of parents who may be at higher risk of experiencing symptoms of complex trauma (e.g., a history of childhood abuse) (coded as ‘0’) and 11 ‘areas of distress’ should be taken forward for discussion with key stakeholders at the second key co-design workshop (Table 1). There was consensus among the working group members that there was a need for an approach to assessment that was flexible and included consideration of strengths in each of these areas.

**TABLE 1:** Domains proposed for inclusion in complex trauma assessment for Aboriginal parents and source tools.

**Proposed domains**	**Source tool**
0. Recognition	None (see text description)
1. Intrusive thoughts	ICD11/DSMV/AAHTQ
2. Avoidance	ICD11/DSMV/AAHTQ
3. Negative thoughts	DSMV
4. Anxiety/reactivity	ICD11/DSMV/AAHTQ
5. Difficulty managing emotions	ICD11/AAHTQ
6. Negative self-beliefs	ICD11/AAHTQ
7. Difficulty maintaining relationships	ICD11/AAHTQ
8. Community disconnection	AAHTQ
9. Loss of identity	AAHTQ
10. Grief and loss	AAHTQ
11. Other personal and cultural impacts	AAHTQ

*ICD11 = 11th edition of the International Statistical Classification of Diseases and Related Health Problems (Böttche et al., 2018; World Health Organisation [WHO], 2018); DSMV = fifth edition of the Diagnostic and Statistical Manual of Mental Disorders (American Psychiatric Association [APA], 2013); AAHTQ = Aboriginal Adaption of Harvard Trauma Questionnaire (Atkinson, 2008).*

### Co-design Workshop Which Included Data Collection for This Research Study

The second key stakeholder co-design workshop in which this research study was conducted was held in Alice Springs, Northern Territory, Australia in September 2018, and the overall objectives and program are reported elsewhere (Chamberlain et al., 2018). We have used recommended standards for reporting qualitative research in this report (O’Brien et al., 2014). Ethical procedures for this workshop were approved by the Central Australian Human Research Ethics Committee (CA-12-3311).

#### Recruitment and Sampling

A flyer inviting people to register for the workshop was circulated through the project email list and community networks. People interested in attending contacted the project team to register. Participation was voluntary and no payments were offered. Travel support was provided for two members of each project partner organization.

#### Researcher Characteristics and Reflexivity

This research study is designed and led by Aboriginal researchers working in collaboration with non-Indigenous researchers to foster safety and relatedness. The research activity was facilitated by an Aboriginal researcher/clinical psychologist (GG), transcribed and coded by an Aboriginal researcher/midwife, with co-development of the preliminary analytic themes and concept map by Aboriginal researchers (CC/GG).

#### Creating a Safe Space

Creating a safe space to facilitate these discussions was a critical element of this workshop. A comprehensive safety protocol was developed based on the discussions in the previous workshop (Clark et al., 2020). Specific strategies to foster emotional and cultural safety for this second co-design workshop included; ensuring a majority of Aboriginal participants, Aboriginal facilitators and presenters leading the discussions, training and preparation, providing information on participant roles and responsibilities prior to the workshop, providing information on managing ‘triggers’ at the start of the workshop, resources to help participants manage distress (e.g., clay modeling, mindfulness coloring, music, refreshments, regular breaks), and finishing the day with an uplifting drumming activity with local school children. Cultural protocols were followed including asking for a Welcome to Country from the traditional custodians (Arrente People). A local traditional healer or ‘Angangkere’ (Ngaanyatjarra Pitjantjatjara Yankunytjatjara Women’s Council and Aboriginal Corporation, 2013) from the Akeyulerre Healing Centre was employed to work alongside a clinical psychologist, to support participants as needed throughout the workshop. As well as enabling a ‘safe space’ for the workshop discussions, these practical processes demonstrate some examples of strategies that service providers may wish to consider in their services.

#### Setting the Scene

The purpose of this research study conducted within the second key stakeholder workshop was to gather views for an assessment tool, not to gather data about participants lives. For this reason the use of story-telling, an important element of Aboriginal culture, was chosen to facilitate discussion about trauma without the need for participants to focus upon, or disclose, personal experiences. Seven senior women from the Ngaanyatjarra, Pitjantjatjara and Yankunytjatjara Women’s Council (NPYWC) joined the group at the start of the workshop and shared a story they had written titled “Tjulpu and Walpa” (Ngaanyatjarra Pitjantjatjara and Yankunytjatjara Women’s Council, 2016) in the Pitjantjatjara language (with an interpreter translating to English). Tjulpu (a bird that sings) and Walpa (the wind) is the story of two girls growing up in two very different contexts and shows how the care a child receives shapes their feelings and behavior. While recognizing challenges some young children experience, this story of hope highlights that wellbeing for young children is possible. Storytelling is important for Aboriginal people, and the women reported that the purpose for developing the book was wanting to share their understanding of the impacts of the past on parents today. Copies of the book were available throughout the workshop on each table. This process helped to ground the workshop discussions in terms of content, culture and community.

### Data Collection

Workshop participants were asked to consider Walpa’s story, which included experiences of violence and neglect. Activity-focused exercises were designed to focus and enhance information gathering (Colucci, 2007). The room was set up so that participants could move between 12 tables, one for each domain. Each table was facilitated by a project investigator who also took notes. Twelve groups of six to eight stakeholders participated in discussions of each of the proposed domains for approximately 5 min, and then moved on to the next table. At each table, participants received a worksheet with a written description of the domain for discussion (see Supplementary File 1 for example) and were asked to:

• Rate (on a five-point Likert scale) how important it is to talk with parents about this domain, e.g., *negative thoughts -* not at all important, not so important, important, very important, not sure.
• Discuss and write down any notes about *why*, *who*, *where* and *how* this domain should be discussed with parents.

### Analysis

Ratings were calculated as percentages of total responses for each of the 12 proposed domains. Response sheets (*n* = 23) submitted without a rating were excluded from the calculations, but the free text comments were included in the qualitative analysis. ‘Important’ and ‘Very important’ responses are reported for each of the 12 domains.

The qualitative approach for this research is grounded theory, which sets out to construct theory from data using a systematic methods and comparative analysis (Chun Tie et al., 2019). Notes from participants and table facilitators were collated and all comments related to w*hy, by whom, where* and *how* discussions about complex trauma should be held were imported into the NVivo software for qualitative data analysis (QSR International Pty Ltd, 2018). Thematic analysis was conducted using iterative steps outlined by authors previously (Braun and Clarke, 2006; Green et al., 2007):

• Immersion in data collection and transcription (CC);
• Line-by-line coding of free text data (CC);
• Creation of descriptive categories (axial codes) and analytic themes using visual tools such as tables and developing a conceptual map (two Aboriginal researchers CC/GG). This inductive process used a constant comparison method to discuss and record notes regarding emergent themes and explore both associations and exceptions to these themes. Color coding was used in a conceptual map to illustrate codes under ‘why, by whom, where and how,’ and shapes to illustrate emergent analytic themes;
• Preliminary analytic themes and supporting quotes were reviewed with the authorship team, to refine and reflect on the themes in relation to the conceptual framework (Chamberlain et al., 2019a).
• Preliminary findings were circulated to seek feedback from workshop participants on the perceived accuracy of the themes, and invite further contribution to the manuscript (anonymously, as a co-author or in the acknowledgments).

## Findings

### Workshop Participants

Fifty-seven key stakeholders, associated with more than 25 institutions across Australia, contributed their expertise to the co-design workshop. The majority were Aboriginal (*n* = 35) and all had extensive experience working with Aboriginal families. A breadth of expertise and depth of wisdom across multiple sectors was present, including community members with lived experience, people working across community organizations, perinatal and family health experts, mental health professionals, senior managers of community and government service organizations, senior members of professional representative and non-governmental organizations, and researchers.

### Perceived Importance of Each Domain

**FIGURE 2:**
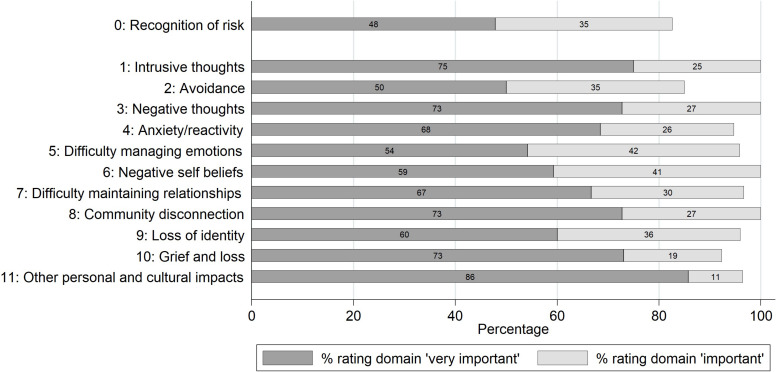
Percentage of responses rating each domain as ‘very important’ or ‘important.’

A total of 297 responses rated the perceived level of importance for each domain. All domains were perceived by the majority of respondents to be ‘important’ or ‘very important.’ All respondents (100%) rated *intrusive thoughts, negative thoughts, negative self-beliefs*, and *community disconnection* as ‘important’ or ‘very important.’ The domains with the lowest percentage rating them as ‘important’ or ‘very important’ were still endorsed by more than four in five participants: *recognition* (i.e., asking people about childhood trauma experiences (83%), *avoidance* (85%) and *grief and loss* (92%) (Figure 2). Participants were also asked if any additional domains may be relevant, but none were identified.

### Summary of Descriptive Coding: Yes Its Important to Talk With Aboriginal Parents About Complex Trauma but ‘How’ This Is Done Is Critical

While there was a high level of agreement among participants that it is important to consider parental experiences of complex trauma, it was more important *why, by whom, where* and *how* these discussions are held. As one participant noted:

“It is definitely important, but HOW you get that info is MORE important. It’s not something that we actually easily/personally recognize it for what it is.” *[Negative self-beliefs]*

**FIGURE 3:**
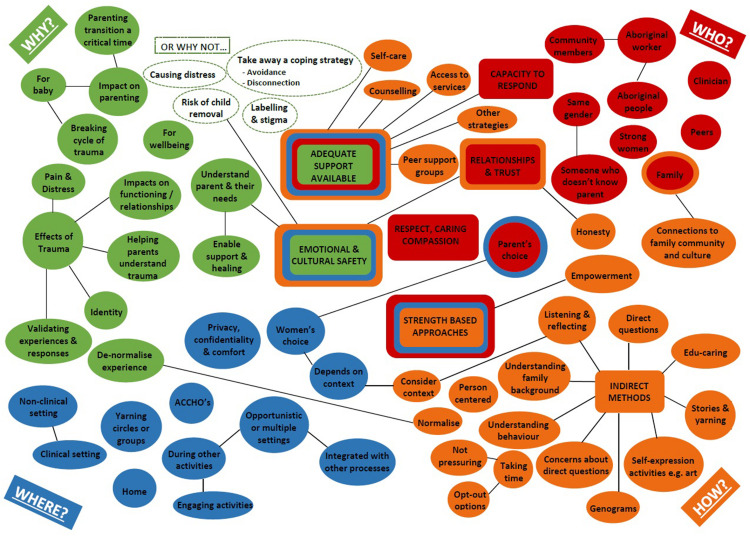
Concept map illustrating key stakeholder perspectives regarding talking about complex trauma with Aboriginal parents.

An outline of descriptive categories (axial codes) are illustrated in Figure 3, related to perspectives of *why* (green), *by whom* (red), *where* (blue) and *how* (yellow) discussions about complex trauma with Aboriginal parents should be held. Some codes were identified in several domains (indicated by rings of corresponding colors), and codes related to strong analytic themes are represented as rectangles.

In describing ‘why’ it is important to talk with Aboriginal parents about complex trauma, participants outlined the serious effects of trauma, pain and distress, and impacts on functioning (including low self-worth) and identity. These impacts relate to each of the domains, including *difficulty maintaining relationships* – with one participant suggesting this was the key question for complex trauma as it relates to all the other domains.

“Having low self-worth and negative thoughts of oneself creates extreme vulnerability to manipulation and control. It significantly impacts daily functioning, relationship maintenance and parenting.” [*Negative thoughts*] “I believe this may be a key screening questions - poor history of relationships. I feel indicates many other risk factors: low self-esteem; trust issues. Risk identified. No safety. Concept of becoming a parent is forming relationship with the infant, partner, mother, community.” [*Difficulty maintaining relationships*] “It is important because your identity is who you are, where you’re from and when you’re feeling disconnected it can often leave you with a sense of loss and grieving.” [*Loss of identity*]

Participants highlighted the importance for baby and the impact on parenting and the parenting transition, and the need to identify existing patterns of unhelpful behavior to break the cycle of trauma.

“Important because as a community we need to work together to stop the cycle of trauma continuing.” *[Recognition]* “Could impact on her parenting, on her, parenting is hard, can bring up a lot of stuff.” *[Intrusive thoughts]* “It is very important to raise this as the negative thoughts and feelings are likely to be transferred to her child…. The excitement of having a child can be embraced rather than the worries, even though the worries exist.” *[Negative thoughts]* “Emotional regulation is key to being parent. You (as mother) need to understand how you manage this in your own self and body because this is a core teaching to your baby. Through nurturing, touch, rhythm, handling, cuddling you are teaching your baby to regulate their body heart rate, temperature, respiratory rate (and therefore emotions). Dis-regulation (flipping your lid) is dangerous for babies.” *[Difficulty managing emotions]*

Participants suggested discussions were also important to help service providers understand the parent and their needs.

“To be able to understand, support and advocate for her needs it is a critical component in establishing a foundation of care, trust, mutual understanding to allow her to tell her story.” *[Recognition]* “Gives the person asking a better idea about what other services need to be involved/referrals. Women are very reserved about expressing these feelings but are sometimes very open to talking about this.” *[Negative self-beliefs]* “Important due to assessing supports available in community outside of service providers. As such vital in developing care plan for mother and child. Also important in understanding location of birth.” *[Community disconnection]*

### Analytic Themes

The following overarching analytic themes, or fundamental pre-requisites, cut across *why*, *by whom*, *where* and *how* discussions about complex trauma should be held with Aboriginal parents in the perinatal setting:

1. **Emotional, physical and cultural safety must be clearly established.**
2. **A trusting relationship with the person talking about complex trauma is critical.** Relational vulnerabilities underpin complex trauma and can impact on readiness to trust. Time is taken to build trust and establish relationships, or involve people who have established a trusting relationship.
3. **Must have the capacity to respond effectively**, including being able to ‘hold the space,’ have time to listen and the skills and support services available. This may involve collaboration with a range of holistic clinical and non-clinical support options.
4. **Incorporate cultural methods of communicating gently and indirectly** to understand the effects of trauma, including the likelihood that parents may be using avoidance as a coping strategy.
5. **Use strengths-based approaches and offer choices to empower parents,** normalize complex trauma responses and affirm hopes and dreams for their family.
6. **Respect, caring and compassion** underpin all discussions and are an essential element for building safety, relationships and trust.

Themes are presented below with a summary of stakeholder perspectives described in text incorporating axial codes and supporting participant quotes. The domain each quote relates to is noted *[italics in square brackets].*

1. **Emotional, physical and cultural safety must be clearly established.**

Having a safe space and/or culturally safe space was the most frequently noted factor in terms of *where* discussions about complex trauma with Aboriginal parents should be held. This linked to comments regarding *how* and *who* should talk with Aboriginal parents about complex trauma.

Privacy, confidentiality and comfort and access to support were also identified as important qualities of *where* discussions are held. Having choice about where and in what context discussions are held were also considered important aspects for fostering safety.

“At the place chosen by woman, private space.” *[Recognition, Avoidance]* “In a safe environment. Spoken in a culturally safe place.” *[Negative thoughts]* “Comfort and safety.” *[Difficulty maintaining relationships]* “In a safe context in which a relationship has been established, in a private setting.” *[Community disconnection]* “Safe, private space once relationship has developed.” *[Loss of identity]* “Safe/private space. Community (beach, park).” *[Loss of identity]* “Safe private space and give some choice/control over venue to patient.” *[Grief and loss]*

Participants noted a range of physical attributes of the places *where* discussions about complex trauma could be held, including clinical and non-clinical settings, home and Aboriginal Community Controlled Health Organisations. Some participants suggested these issues might be discussed in yarning circles or groups, while others emphasized the need for privacy, which reinforces the need to consider individual choice and preferences. Some participants also suggested discussions could be opportunistic and held during other [engaging] activities.

“Sometimes this can be good sharing in yarning circles with other women from their Aboriginal group or community.” *[Recognition]* “At Aboriginal birthing service - ask when they have relationship.” *[Recognition]* “During transport, family outings, anywhere the clients feel safe and can ensure privacy.” *[Recognition]* “Could be clinical setting, home environment if safe.” *[Negative thoughts]* “Some women may find it hard to open up in a ‘clinical setting’.” *[Difficulty maintaining relationships]* “Time tricky, e.g., if antenatal appointment - limited time. Home visit better.” *[Grief and loss]* “Community centers, neutral spaces, clinic.” *[Other personal and cultural impacts]* “Remove formalized structures in environment (i.e., in the car).” *[Other personal and cultural impacts]*

While a number of respondents highlighted the importance of parent choice in deciding *who* they would like to talk to, some respondents suggested more specifically that it should be someone who doesn’t know the parent, and others felt it was important to have someone of the same gender talking about trauma.

“Important for women to engage with women.” *[Recognition]* “Men to men - Indigenous men or cultural Elder.” *[Difficulty maintaining relationships]* “Perhaps someone who does not know your relationships to take on an objective perspective.” *[Difficulty maintaining relationships]*

There were mixed opinions about whether the person talking about trauma should be an Aboriginal worker or other clinician. Some respondents talked about offering an Aboriginal worker with an option of speaking to a non-Indigenous worker.

“Sometimes community workers aren’t best placed if they know the person.” *[Recognition]* “Perhaps a multidisciplinary team could be introduced in the early days.” *[Intrusive thoughts]* “Aboriginal worker with option of non-Aboriginal.” *[Negative thoughts]* “Long term case manager, or counselor, or group therapist.” *[Negative self-beliefs]* “An Aboriginal worker to address. Consider seeking advice from an Elder.” *[Loss of identity]*

Several respondents suggested discussions could be held with non-health staff, including Aboriginal people, a community member, family, peers, and strong women.

“Strong women in community.” *[Recognition]* “The community is responsible for all children.” *[Recognition]* “Elders in community will listen, just listen and tell her stories to make her strong so she can let go and become free and become a better mother.” *[Intrusive thoughts]* “Nannas, Aunties,” *[Intrusive thoughts]* “Strong young mums and Elders in the community.” *[Avoidance]* “Peer group - young mums to give security to allow you to open up.” *[Avoidance]* “Peer and/or friends, strong role model/mentor.” *[Negative thoughts]* “Extended family, with someone trusted, safe.” *[Difficulty managing emotions]* “I often believe mothers, grandmothers and family or close friends.” *[Negative self-beliefs]* “Choice of Aboriginal person.” *[Grief and loss]* “Families, community organization running loss and grief talking circles.” *[Grief and loss]* “Hopefully the Big Aunty or other strong family member, clinicians, support workers, nurses, counselors.” *[Grief and loss]*

Other suggestions included neighbors, school teachers, people who are ‘young in mind’ ‘thoughtful,’ and ‘not too grievous themselves.’ Participants expressed concerns about these discussions being conducted within child protection services, and triggering harmful responses. Participants also discussed possible issues with disclosing childhood trauma with family present in the room.

The importance of safety and cultural security were reinforced in the comments workshop participants made about *how* to talk with parents about complex trauma.

“Once the opportunity has been created to have a safe place then the avoidance can be named, gently, to provide a respectful environment to explore it or to start a referral pathway.” *[Avoidance]* “Reflective, culturally safe.” *[Avoidance]*

2. **A trusting relationship with the person talking about complex trauma is critical.**

Trust and relationships were considered a fundamental pre-requisite for talking about complex trauma and were linked to being culturally safe.

“Relationship and trust important to feel safe” *[Recognition]* “Need to build the relationships to be able to have the conversation not just tick the box” *[Recognition]* “Trusted person - whoever that may be (Aunty, health worker, could be a man if had trusted relationship).” *[Intrusive thoughts]* “Important to develop relationship/trust just as this is not an easy question to ask on first meeting.” *[Anxiety/reactivity]* “Trusted person, who can help with strategies and safe relationship - long term work.” *[Anxiety/reactivity]* “Someone trusted - this sort of information will naturally become evident so the trusted person can gently empathize and draw awareness to as opposed to ask directly and abruptly.” *[Difficulty maintaining relationships]* “Needs trust. Won’t divulge without trusting relationship.” *[Grief and loss]*

The importance of trust, relationships and honesty were reinforced in comments related to *how* to talk with parents about complex trauma.

“They should be able to express stuff when they feel empowered and safe. They may feel afraid of reprisals if they speak out so might lie and so they might not receive the right care.” *[Recognition]* “Acknowledging the practitioners knowledge gaps is important to be sensitive and realistic. If the question is asked the answer needs to be able to be managed well so the mother feels safe.” *[Intrusive thoughts]* “Those trying to help can create trust by understanding those behaviors for what they are.” *[Avoidance]* “This sharing from the subject may take time: trust developing; safe space…; ongoing relationship will help facilitate their involvement with services. Shame is/can be such an inhibitor of sharing such personal beliefs. May take many sessions to overcome this. Perhaps shame can be reviewed more closely and dissected.” *[Negative self-beliefs]* “Important to build a SOLID relationship prior to asking or eluding to this topic.” *[Negative self-beliefs]* “May be asked in layers as relationships develop - understanding takes time.” *[Community disconnection]* “To support any Aboriginal person, particularly a young mum, it is critically important to engage with the notion of personal and cultural identity as a foundation of strength, belonging, spirituality and safety.” *[Loss of identity]* “It’s important to find this out in a culturally appropriate way because you need to develop trust firstly.” *[Other personal and cultural impacts]* “Connection and trust is very important.” *[Other personal and cultural impacts]* “How you ask the questions - be upfront and honest about why asking.” *[Other personal and cultural impacts]*

Considering connections to family, community and culture was also identified as an important factor in *how* discussions about trauma should be held, specifically for enquiries related to the domains of *community connection*, *loss of identity* and *personal and cultural impacts*. Some participants suggested that involving family and/or community members could be helpful in having someone who understands the parent’s experiences and enabling local support. However, other participants thought that involving family and community members could be problematic, especially where trauma has been experienced in the family of origin and/or where the context may raise concerns about confidentiality.

“The work on community is also to talk about disconnection from family. I have found that connections are generally strong to community and culture. Connection in this way is central to healing and strength - something that can be drawn from and built on.” *[Community disconnection]* “Look through intake or referral notes from other services - seek info as to who the connections are (e.g., friends, pets, schooling, not just blood family). Re-establish some roles to identify with (e.g., kind Aunty or strong mum or caring for my pet).” *[Loss of identity]* “OR maybe the client may not want to be involved in culture because of how complicated family/culture life is. Be sensitive in asking.” *[Loss of identity]* “Risks- not from urban setting or community Elder not able to help because of conflict of families’ history.” *[Loss of identity]* “Could come about when asking a woman about her support networks, she might reveal the gaps in her network.” *[Grief and loss]* “Most important. Culturally sensitive ways to ask as some people have rejected culture, rejected personal cultural networks for reasons.” *[Other personal and cultural impacts]*

3. **Capacity to respond effectively is essential.**

Having the skills to respond appropriately and having support available were also considered very important qualities with regard to the question of *who* should talk to Aboriginal parents about complex trauma.

“People who have trust and skills to handle it well.” *[Recognition]* “Needs to be a conversation with someone who is equipped to take in the information and use it appropriately to assist the individual to process and manage” *[Recognition]* “You can’t ask if you don’t have the right supports.” *[Recognition]* “Someone who the person trusts, chosen by them, AND who has trauma-informed practice knowledge, i.e., best NOT to ask (re-traumatize/shame) if we’re not able to support the answer?” [*Difficulty maintaining relationships]* “Someone she knows and trusts, and hopefully someone with the training to explore these areas.” *[Other personal and cultural impacts]* “Person who is asking questions would ideally be able to provide this psychological support/been present in the woman’s care previously and thus be a trusted person. Otherwise person able to introduce to other support?” *[Other personal and cultural impacts]*

Other suggestions included being able to ‘hold the space’ and provide appropriate responses and support strategies, peer support groups, counseling, access to services, self-care, providing coping strategies, mastery activities and other strategies (including ceremony) that help.

“Slowly, gently and only when there is the possibility of being able to ‘hold a space’ and deal appropriately with the answer.” *[Avoidance]* “Peer support groups would be ideal, and counseling.” *[Negative thoughts]* “Have a supportive network, activities, programs etc to offer them ready to offer.” *[Anxiety/reactivity]* “Counseling, safe work group.” *[Difficulty managing emotions]* “Create activities - mastery - that challenge those beliefs.” *[Negative self-beliefs]* “Community workshops for children to provide positive good experiences. Council in community putting on family fun days. Fashion parades for kids. Kids telling stories.” *[Difficulty maintaining relationships]* “Ceremony - to settle the loss. Ceremony to welcome the baby - new life.” *[Grief and loss]* “Strategies in place to deal with answers, support for woman and person asking.” *[Other personal and cultural impacts]* “Important to introduce strategies and care to manage symptomology and assist in attachment between mother and child. Strategies the mother can retain and teach child. Practitioner led in what strategies/whether they are wanting assistance.” *[Other personal and cultural impacts]*

4. **Cultural methods of communicating gently.**

While many participants noted the importance of talking with Aboriginal parents about complex trauma, there were concerns about asking direct questions (particularly in relation to childhood experiences) and extensive comments about the importance of having discussions in a way which was respectful, caring and understanding. Participants also discussed how indirect methods of enquiry that are less confronting and more culturally appropriate could support understanding.

“Not to be direct questions. Information will come out during yarning once relationship has been built.” *[Recognition]* “I like the way the discussion is introduced on the other side [of this sheet] - providing people with the option of talking about it without imposing the discussion on them.” *[Recognition]* “Less questions. Yarn. Build up relationship/respect.” *[Intrusive thoughts]* “Asked in a way that is not interrogating or naming, shaming, blaming. Education and awareness making in mind.” *[Difficulty managing emotions]* “This is important to know and address but even without asking about it, you would seek to address such feelings/thoughts through the therapeutic relationship.” *[Negative self-beliefs]* “Someone trusted - this sort of information will naturally become evident so the trusted person can gently empathize and draw awareness to, as opposed to ask directly and abruptly.” *[Difficulty maintaining relationships]* “It is important to find out the information but I don’t know that it is important to direct the question to her.” *[Other personal and cultural impacts]* “Gently - have things happened that you wish didn’t happen to you? Yarning back and forth. Things you want for your baby that are different from what happened to you.” *[Other personal and cultural impacts]*

Participants also talked about the importance of helping parents to understand the effects of trauma as parents may not be aware of these. This consideration also relates to avoidance, as some parents may not feel ready or able to cope with the feelings of distress associated with understanding complex trauma. Participants talked about avoidance as a barrier to enabling support and healing, wellbeing and promoting safety, but also recognized avoidance is an important coping strategy. It was noted that care was needed not to undermine this coping strategy, particularly in the absence of effective support. While there was no ‘one right way’ to talk with Aboriginal parents about complex trauma, some suggested strategies for ‘how’ to talk with parents included using cultural ways of talking and listening. These include: ‘educaring’ which is premised on each person being able to know themselves and be capable of making life choices to enhance growth (Australian Institute of Family Studies, 2012); ‘dadirri’ which is a form of contemplative deep listening and quiet still awareness (Atkinson, 2002; West et al., 2012); family mapping (genograms) and self-expression activities (e.g., art); story-telling and ‘yarning’ exemplified in the use of the Tjulpu and Walpa story in this workshop. Participants explained how these strategies could help people to understand patterns of intergenerational trauma and identify elements in their own experience. These gentle and indirect approaches are more consistent with Aboriginal ways of having these kinds of conversations.

“It may not be important to ask her whether she avoids things, but it is important that the community and service providers should understand that the behaviors exhibited are avoidance mechanisms. She herself may have no comprehension that she is avoiding reminders. The behaviors might not be overt.” *[Avoidance]* “May not recognize the triggers - can be smells, sounds. Also may not reveal as individual may not be aware that they avoid.” *[Avoidance]* “Can also be caught off guard when avoidance doesn’t work - hard to avoid every trigger - sometimes hard to know what they are.” *[Avoidance]* “They need to talk it out and understand that the traumatic events are external to them and not who they are as a person. They may not understand why they feel what they feel and being able to talk about it can help them contextualize it. To understand that when they have been told it is their fault, that they are wild etc, that it is not true. They don’t understand this unless they have been told. So they need someone to talk about it with them.” *[Negative thoughts]* “Important to identify - how is it affecting life/wellbeing/health/parenting? How can it be worked through/overcome or managed in adaptive and healthy ways?” *[Grief and loss]* “Educate FIRST about trauma - and then develop trust and wait until they are ready to talk. Need to educate as some people don’t know they are in violence. Everyone is exposed trauma - so unpacking what that means and simplify it and then be able to hold that space. Would take time to think about it and whether to trust.” *[Recognition]* “Supporting their narrative. Genogram and storytelling.” *[Recognition]* “Genograms really great place to start yarning - but get direct information about family structure and how they grew up.” *[Recognition]* “Noticing the physical symptoms and the triggers for this (i.e., preceding event). Saying “I noticed that this happened when we did this” and slowly moving toward identifying the anxious symptoms.” *[Anxiety/reactivity]* “Generating a mindfulness around healthy spaces for growing baby and the negative impacts we can create in our emotional response (e.g., anger, shouting, fighting). This creates distress for baby. Find healthy ways to express difficult emotions.” *[Difficulty managing emotions]* “Indirectly - explaining through story, other’s stories. Using art pictorial resources.” *[Negative self-beliefs]* “Yarning is good so they can feel safe and secure that someone is there to talk to them. Some get shame - just put words in plain English that’s my way of asking clients in a cultural way.” *[Difficulty maintaining relationships]* “A Kinetic family diagram is a less confronting way to explore quality and density of social support system. Always ask from strengths perspective: what strategies keep you strong? Who do you go to/talk to when under pressure?” *[Difficulty maintaining relationships]* “As well as talking, other non-verbal ways. Draw family and mob: where baby sits; imagine baby in that ‘family tree’ and place baby and self.” *[Community disconnection]* “This question may be asked in the form of a genogram or other narrative around family, friendships and kinship - visual or otherwise.” *[Community disconnection]* “Use of stories, drawing, ways of creative expression, not just words.” *[Grief and loss]* “Explain and link to emotions and actions in persons experiences. Educare - why are we asking?” *[Other personal and cultural impacts]*

Participants raised a number of concerns about asking parents direct questions about trauma and gave reasons why not to do this. While recognizing avoidance and disconnection as symptoms of distress, participants also perceived these to be an important way of coping with trauma. Participants emphasized the importance of not undermining existing coping strategies without offering alternative coping strategies). Other risks identified by participants included loss of safety, risk of child removal, labeling and stigma, causing distress, inaccurate disclosure and a lack of adequate supports available.

“You need to be careful asking because you can make her feel more vulnerable and judged.” *[Recognition]* “Child protection ‘risk’ sometimes makes it unsafe for women to bring up.” *[Recognition]* “Avoidance is a coping strategy - be careful what we take away.” *[Avoidance]* “This is long term work, it’s not for screening.” *[Avoidance]* “I think asking this question may make them feel responsible and a sense of failure if they are not successful at ‘controlling’ themselves. They are reacting to external forces and I think we need to make sure that we don’t make them feel worse.” *[Difficulty managing emotions]* “It is private family business - inappropriate to discuss. Is up to family if they want to discuss. Working in this space we must (perhaps) assume that everyone we meet is/has experienced grief and loss and unresolved pains. So perhaps we don’t need to ask? At the least, ONLY ASK IF YOU HAVE THE THERAPEUTIC TOOLS TO BEGIN HEALING.” *[Grief and loss]* “She may have difficulty providing an honest answer and that may impact her care pathway.” *[Other personal and cultural impacts]*

Participants also highlighted that gentle approaches were critical to how discussions were held. Taking time and not pressuring parents to answer questions, with opt-out options were also seen as critical.

“Safely. Not confronting. Gently.” *[Difficulty managing emotions]* “Ensure they feel safe/secure. Allow the client to have time to talk.” *[Recognition]* “Name it, but respectfully and don’t force the person to overcome, let them take their own time.” *[Avoidance]* “When they are ready to talk, not pressuring her to talk, placing no judgment.” *[Anxiety/reactivity]* “Being available and taking the time and gauging responses and reactions.” *[Difficulty maintaining relationships]* “Aboriginal culture relationships take time to establish. Take interest in them; need time.” *[Other personal and cultural impacts]*

There were many suggestions about the importance of listening and reflecting, understanding behaviors, considering context, and being flexible and person-centered in *how* to talk with Aboriginal parents about complex trauma.

“Need to assess/understand the behaviors and what they indicate.” *[Avoidance]* “A lot of this information can be gleaned via mental state examination, i.e., careful observation and attunement of the interviewer with affect of the person being interviewed.” *[Negative thoughts]* “Observation by interviewer can reveal mood/affect lability.” *[Difficulty managing emotions]* “A lot of emotions are also physical, so allowing the mum to notice what was happening for her cognitively and physically when she was getting angry. If she gets mad at you in the therapy session, address it later when she is more calm.” *[Difficulty managing emotions]* “What it is feeling like for him - intensiveness. Responses, silences, non-verbal cues, retracting. Using emotional intelligence with sensitivity and care.” *[Difficulty maintaining relationships]* “The setting/context is very important - it could be very distressing/shaming to question this. How do you ask without re-traumatizing?” *[Difficulty maintaining relationships]* “Person-centered approach.” *[Difficulty maintaining relationships]* “In all situations - Being seen-heard-given value to. Being ‘Aboriginal’ is multi-dimensional multi-layered multi-storied.” *[Loss of identity]* “Non-verbal ways of recognition.” *[Other personal and cultural impacts]*

5. **Strengths-based approaches and offer choices to empower parents.**

A number of participants noted the importance of parent choice in deciding *who* they would prefer to talk to about issues related to complex trauma.

“Give choice/control and some agency to the woman to decide when and who she shares this with.” *[Recognition]* “Not necessarily one person. You might want to talk to someone disconnected from your community because there will be little attachment or consequence. Alternatively, you might want to speak to someone deeply connected who will have a better understanding of your experience.” *[Intrusive thoughts]* “Give choice - gender, Aboriginal/non-Aboriginal, professional/personal.” *[Other personal and cultural impacts]*

Some participants suggested that trauma may not always be recognized because of ongoing experiences of violence that may be ‘normalized’ in some Aboriginal communities. They suggested it is important to help parents understand that experiences of violence were not normal and to validate their experiences and responses. However, while participants talked about the need to ‘de-normalize experiences of violence,’ they also emphasized the importance of ‘normalizing’ trauma responses as natural defensive mechanisms to threats.

“Trauma is normalized in many communities, may not be aware that their stories are trauma. Therefore need to ‘hold the space’ for that realization.” *[Recognition]* “To validate and normalize her experience.” *[Negative self-beliefs]*

Using strengths-based approaches that foster empowerment and recognize that complex trauma responses are difficult but also natural responses to extreme, often multiple traumatic experiences was also seen as important. These understandings link to previous comments regarding choice and control.

“Come from a strengths-based place (Hopes and dreams, survival strategies, kept herself safe)” *[Recognition]* “Normalizing - quite common when have experienced those things.” *[Intrusive thoughts]* “Need to build strength. Come from a position of hope, strength, power.” *[Avoidance]* “This in a way can empower the mother to make her own choices to seek help when SHE is ready.” *[Avoidance]* “Highlight strengths. Protective response - making links, fight responses, etc. Help people not to feel crazy.” *[Anxiety/reactivity]* “Normalize the response. Not going crazy - others feel this way” *[Anxiety/reactivity]* “Build on self-worth, confidence, make stronger to help her feel she is in control of her own wellbeing.” *[Negative self-beliefs]* “When family relationships are/have become unhealthy - establish possibility of healthy reliable NURTURING experiences.” *[Difficulty maintaining relationships]* “Share through “doing.” Therapeutic healing process/rituals - to tap into the stories of loss and acknowledge it, express it etc. Do not be scared to speak of those who have passed, e.g., if Grandma was here - what advice would she give you? Draw on her strengths rather than focus on the loss.” *[Grief and loss]* “Set strength-based foundations in the conversation. Her preferred narrative - hopes, dreams, ambitions - and then find a way of respectfully enquiring around her concerns, worries - dominant narrative.” *[Negative thoughts]*

6. **Respect, caring and compassion.**

Respect, caring and understanding (or compassion) were seen as important qualities for people talking with Aboriginal parents about complex trauma.

“Somebody that is caring and understanding.” *[Avoidance]*

Ensuring parents are feeling supported and not judged, showing compassion, kindness and care and being mindful of reactions were emphasized as important aspects of *how* to talk with Aboriginal parents about complex trauma.

“Mindful of how you react to what she says.” *[Intrusive thoughts]* “Not judging, gentle, taking time, telling stories, kind.” *[Anxiety/reactivity]* “In a non-judgmental way, respectful.” *[Negative self-beliefs]* “Ask in a manner that she will feel someone is there for her and to be listened to.” *[Negative self-beliefs]* “To be asked in a caring and helpful way.” *[Loss of identity]*

Some participants noted that assessment of some of the domains (e.g., in the area of personal and cultural impacts) could be integrated with other processes, such as the Edinburgh Postnatal Depression Scale and the Aboriginal Health Check, and some questions may be unpacked in others. There were also some wording suggestions, and these were considered by the assessment working group and incorporated into the assessment tool language development.

## Discussion

This study aimed to understand perspectives of predominantly Aboriginal key stakeholders involved in supporting Aboriginal parents experiencing complex trauma regarding; the importance of proposed domains for inclusion in an Aboriginal Complex Trauma Questionnaire, and why, by whom, where and how these discussions should be held. There was consensus among the 57 predominantly Aboriginal co-design workshop participants that it is important to recognize Aboriginal parents who are experiencing complex trauma. However, there were concerns about where, with whom and how discussions with Aboriginal parents should be held, and these considerations were viewed as more important than the assessment items themselves. In general, there were more comments and greater consistency regarding the ‘qualities’ of who, where and how discussions should be held, rather than specific ‘attributes.’ Key considerations included: ensuring emotional, physical and cultural safety; building a relationship and trust; having capacity to respond effectively; incorporating gentle, indirect cultural methods of communication; using strengths-based empowering approaches and employing respect, caring and compassion. These issues align with the core values of the HPNF project’s conceptual framework (Chamberlain et al., 2019a), including; safety, empowerment and choice, trustworthiness and relationships, collaboration, culture, holistic care and compassion.

Our findings are similar to those previously identified by British (Yapp et al., 2019) and Australian women in pregnancy (Rollans et al., 2013), that it is acceptable to ask about mental health but that this can cause discomfort and distress, particularly where there is a history of abuse. Women noted that the approach used by the person asking impacted on their disclosure, and that they wanted to be asked clear questions, have sufficient time to discuss issues, and receive responses which were validating and well-informed (Yapp et al., 2019). A study in the United States similarly reported that many women might not characterize their experiences as ‘trauma’ and prefer the use of less stigmatizing words such as ‘hurt’ (White et al., 2016). The authors described five themes required for ‘safe’ trauma inquiry including: a clear definition of trauma, a clear purpose for inquiry, reassurance that inquiry was routine, confidentiality, and the mention of helpful resources other than psychiatric therapy (Seng and Petersen, 1995; White et al., 2016). Some who felt that the question should be asked, reported that they felt unable to bring up the topic themselves (Seng and Petersen, 1995), suggesting these discussions may help create safety if done in the right way. Women elsewhere have also reported feeling more comfortable with ‘psychosocial assessment’ after a ‘period of familiarization’ (Reid et al., 1998). The importance of the therapeutic relationship, understanding what clients are not saying, and variations of how and when to discuss experiences of trauma were similarly identified in a review of antenatal screening for intimate partner violence (LoGiudice, 2015).

There are several strengths of the research outlined in this paper, including the breadth of expertise, small group sessions, safe processes and community leadership. However, there are several limitations. First, in order to make efficient use of participant’s limited time, the data collection methods involved direct questions and relatively quick written responses, rather than more in-depth qualitative interview methods. As a result, the data collected have more ‘breadth’ than ‘depth.’ Second, workshops were relatively structured, which may have led to predetermined themes emerging. Third, participants were predominantly service providers, policy-makers and researchers, who have provided advice on strategies for subsequent discussions with Aboriginal parents. Fourth, while participants were asked if ‘other’ domains should be included and none were identified, it is possible that other domains may be relevant.

These findings have important implications for population-based screening policy in relation to complex trauma. Ensuring the benefits outweigh the harms is a standard pre-requisite to any routine screening or assessment process (Wilson and Jungner, 1968). Processes to ensure these pre-requisites are rigorously met have been identified as critical for Aboriginal people (Scrimgeour, 1996). The views shared by workshop participants made it clear that the primary purpose of talking about complex trauma was to enable support for parents who may be experiencing significant levels of trauma-related distress. As such, discussions should aim to foster safe and responsive engagement that supports and validates parents. There was strong consensus that these discussions should not be associated with child protection services or making standardized assessments of ‘risks’ to child safety. Practitioners in the Aboriginal perinatal sector need to always remain cognizant of Australia’s historical and ongoing practices with regards to removal of Aboriginal children from families, and parents’ subsequent fears. There is a need for culturally responsive protocols and proactive engagement strategies to support parents who may be experiencing significant levels of distress, with focus on support, and culturally guided steps to follow should there be significant concerns for women’s, children’s or men’s safety. Participants also reinforced the importance of including strengths-based assessment of resilience in any assessment of complex trauma-related distress to foster empowerment and counteract the dominant deficit discourse in Aboriginal health (Fogarty et al., 2018).

Findings from this study have several implications for practice. First, human services and child welfare/family support systems play an important and challenging role in supporting vulnerable families. Their intersection with the health system and perinatal sector can be particularly complex, differ from state to state, and often highlight systemic issues (Bromfield et al., 2014). In relation to Aboriginal families, service providers need to be clear about their processes for managing this intersection so as to maintain the relational integrity of their care for parents and children without using the system to enforce compliance, undermine established relationships or misrepresent Aboriginal parents’ complex trauma symptoms and coping strategies, such as ‘avoidance.’ Strategies consistent with the national trauma-informed care principles of safety, transparency, choice, collaboration and empowerment (Kezelman and Stavropoulos, 2012) are likely to be more effective and substantially less harmful for Aboriginal parents.

Building relationships and trust is vital in the context of complex trauma, sometimes referred to as relational trauma, and these findings have critical implications for practice. One workshop participant noted relationships could be the key question in relation to complex trauma. Parents experiencing complex trauma may have had a childhood history of previously disclosing abuse and not being believed by family, other community members or service providers, which impacts on trust and a sense of connectedness, which in turn can impact on and compound experiences of grief and loss. Growing evidence supports continuity of carer perinatal care models to enable relationships to be developed, including comprehensive multidisciplinary models (Hickey et al., 2018) which have demonstrated significant improvements in birth outcomes (Kildea et al., 2019). Knowing when a trusting relationship has been established can be a challenge in the context of busy perinatal clinical settings. However, it is critical that all practitioners are able to provide safe care through capacity to listen deeply and talk about sensitive issues gently and with respect, caring and compassion.

A third implication for practice is having capacity to respond to parental disclosure of complex trauma requires practitioners to have expertise, good collaborations and referral networks. Prior to colonization and medicalization of birthing, there were sophisticated networks to support Aboriginal community members, including parents during this time (Ramsay, 2014). Girls were identified as having potential for a midwifery role in the community; and were mentored through a comprehensive and systematic process to develop technical skills and emotional intelligence, which some argue is the basis of developing ‘wisdom’ (Chamberlain et al., 2016). There was also a supportive network of senior midwives, Elders and others in the community to support parents as needed. Re-claiming and understanding this ancestral wisdom is critical from an Aboriginal standpoint. It is the foundation for fostering the gentle, expert and wise support required for working effectively with vulnerable Aboriginal parents experiencing the multiple and serious effects of complex trauma during the critical parenting transition.

Providing parents with information about trauma could help people to understand patterns of intergenerational trauma and identify elements in their own experience (Australian Institute of Family Studies, 2012). Information may be able to be provided within an individual or group setting, such as a ‘yarning circle.’ This is important as experiences of trauma are sometimes ‘normalized’ within communities (Brush et al., 2018), and parents may not understand how they have been affected by their childhood. There is a need to ‘de-normalize’ parent perceptions of childhood trauma experiences, and at the same time ‘normalize’ perceptions of complex trauma responses. This entails helping parents understand that the feelings of distress they are experiencing now are natural responses to their previous experiences of childhood trauma. This process is consistent with the Power Threat Meaning Framework (PTMF) [28], which essentially reframes behaviors related to complex trauma as normal self-protective responses to threatening situations, rather than pathological deficits. The question becomes “What has happened to you?” rather than the diagnostic framing of ‘What is wrong with you?” [28]. Providing information and offering parent choice may also empower parents to navigate their way safely through perinatal care, by enabling them to assess when and where they feel safe and decide who they trust. This ‘educaring’ approach frames the helping professional working in a natural, non-intrusive way akin to ‘*a midwife, an attendant at the birth of new knowledge, new understanding of the Self.*’ P 211 (Atkinson, 2002).

Aboriginal ways of communication and interpersonal styles of engagement, such as yarning and dadirri (Atkinson, 2002; West et al., 2012), were considered appropriate for discussions regarding complex trauma by workshop participants. They were seen as less direct and confronting than many clinical assessment approaches. These conversations often occur incidentally and opportunistically during other activities (e.g., traditional activities, creating art, driving or cooking), which enhances safety by allowing opportunities for people to raise sensitive issues when they feel safe and ready. It is also consistent with traditional midwifery approaches of working ‘side by side’ and ‘with women’ through the transitions of pregnancy and birth, and offers a safe way of providing support in a non-stigmatizing and empowering way (Bradfield et al., 2019).

Findings from this research study will inform the subsequent phases of the broader HPNF project, which includes discussions with Aboriginal parents about preliminary questions to include in an Aboriginal complex trauma assessment tool, another key stakeholder workshop to refine strategies to ensure safe ‘recognition’ of parents experiencing complex trauma, and psychometric evaluation of preliminary items for an Aboriginal complex trauma assessment tool. There is an urgent need for research to support decision-making regarding awareness, recognition, assessment and support for Aboriginal parents experiencing complex trauma in the perinatal period. This includes: development of resources and processes to increase *awareness* regarding complex trauma for parents, community members and service providers; acceptable and feasible recognition and assessment processes; and a comprehensive evaluation of innovative strategies to better support parents; so that these learnings can be shared across the sector. We suggest any evaluation strategies should include short reflective cycles (e.g., action research) to detect and manage any unforeseen risks and consequences.

## Conclusion

The majority of key stakeholders suggest that talking with Aboriginal parents about the effects of complex trauma is important. However, *how* these discussions are conducted is fundamental. Ensuring emotional and cultural safety was considered absolutely paramount, enabled through building relationships and trust, having capacity to respond appropriately and provide support, and incorporating culturally appropriate less direct approaches such as yarning and dadirri. Using strengths-based approaches, offering choice to empower parents, and showing respect, caring and compassion were considered vital. It is essential to ensure that these factors are enacted before talking with Aboriginal parents about complex trauma. Otherwise, there is a high risk that harms such as stigma, re-traumatization and potential family disruption, may outweigh any potential benefits of talking with Aboriginal parents about complex trauma, contravening international population-based screening criteria.

## Data Availability Statement

The raw data supporting the conclusions of this article will be made available by the authors, without undue reservation.

## Ethics Statement

The studies involving human participants were reviewed and approved by Central Australian Human Research Ethics Committee. The patients/participants provided their written informed consent to participate in this study. Written informed consent was obtained from the individual(s) for the publication of any potentially identifiable images or data included in this article.

## Author Contributions

CC is the Principal Investigator for the project who conceived the study, developed and submitted protocols and ethics, and drafted the manuscript. GG, DG, FM, YC, CA, TH, HM, HH, SB, and JN are investigators who contributed to research and workshop design. All authors contributed to data collection in assisting with co-facilitation of workshop. GG led the workshop facilitation and conducted preliminary data analysis with CC. DG developed the conceptual map. DG, FM, YC, NR, CA, TH, HM, TE, HH, SB and JN assisted with workshop planning and facilitation, interpretation of data, refining themes and revision of several drafts, including the final manuscript.

## Conflict of Interest

CA was employed by the company We Al-li Pty Ltd. The remaining authors declare that the research was conducted in the absence of any commercial or financial relationships that could be construed as a potential conflict of interest.
