# Supplementary material for: Community Perspectives of Complex Trauma Assessment for Aboriginal Parents: ‘Its Important, but How These Discussions Are Held Is Critical’
Source: Front Psychol. 2020 Sep 15;11:2014. doi: 10.3389/fpsyg.2020.02014 (PMC7522325; doi:10.3389/fpsyg.2020.02014)
Supplement: Supplementary file 1 [file Data_Sheet_1.docx]

**Supplementary File 1: Sample area of distress discussion sheet**

1. **Recognition**

**To provide the young woman from the Walpa story with the best possible care, how important do you feel it is to ask her in a personal and culturally respectful way whether she’s been around any personal or community violence?**

**Please circle the word to show how much you agree or disagree with the need to ask her about this.**

**Not sure Not important at all Not so important Important Very important**

**Example conversation: *‘Becoming a parent can be one of the most rewarding and important things that ever happens in our life. Sometimes first time parents can also struggle with their own issues though, and we know that being around things like community violence can sometimes really affect people and even make their parenting experiences much more challenging. But we also know that becoming a parent is a great opportunity for healing. Would you feel comfortable if I talked a little about trauma and healing? (If the answer is yes) ‘I wanted to ask if you have ever seen or been around any violence in your community - for example physical violence, emotional abuse, or sexual violence - that you feel has affected your wellbeing in a negative way? If you have, I’m not going to ask you about those experiences. Instead, I’d like to know if you’d be interested in getting support around healing and knowing more about how being around things like violence can affect us in different ways.’***

**Please feel free to write WHY you think it is or isn’t important to ask the young woman about this - and if important- WHO, WHERE or HOW the young woman should be asked about potential exposure to trauma.**
